# Malignant Transformation of an Ovarian Endometrioma during Endometriosis Treatment: A Case Report

**DOI:** 10.1155/2018/6210172

**Published:** 2018-09-04

**Authors:** Hiroaki Takagi, Emi Takata, Jinichi Sakamoto, Satoko Fujita, Masahiro Takakura, Toshiyuki Sasagawa

**Affiliations:** Department of Obstetrics and Gynecology, Kanazawa Medical University, School of Medicine, Japan

## Abstract

Dienogest (DNG) is considered to be effective against ovarian endometrioma (OMA). We report a rare case of OMA transformation to ovarian cancer during long-term endometriosis treatment with a periodic administration of a gonadotropin-releasing hormone agonist (Gn-RH agonist) and DNG. The patient was a 41-year-old Japanese woman. OMA and adenomyosis of the uterus were revealed via computed tomography. Consequently, she underwent conservative treatment without undergoing surgery because her overall status was poor. She received cyclic therapy (Gn-RH agonist and DNG) for approximately eight years. However, she reported lumbago and underwent close medical examination at our hospital after about eight years of treatment. Under the suspicion of malignant transformation, she underwent surgery. The pathological diagnosis was clear cell carcinoma of the right ovary (stage 2B). After surgery, she received six courses of chemotherapy (conventional TC). No evidence of disease was observed after chemotherapy. Our findings suggest that malignant transformation of OMA can occur during DNG treatment. Since the delayed detection of ovarian cancer greatly affects the prognosis, women older than 40 with OMA are encouraged to undergo regular check-ups every few months.

## 1. Introduction

Currently, Japanese women are marrying later in life, which greatly increases the number of menstruations and the risk of endometriosis [1]. Treatment with dienogest (DNG) has helped reduce pelvic pain and improved quality of life in patients with endometriosis [2]. In addition, DNG is expected to reduce the size of an ovarian endometrioma (OMA) [3], and the treatment of endometriosis has been expanded. Herein, we report a rare case of OMA transformation to ovarian cancer during long-term endometriosis treatment with the periodic administration of a gonadotropin-releasing hormone agonist (Gn-RH agonist) and DNG.

## 2. Case Presentation

A 41-year-old Japanese woman (gravid: 0; para: 0; height: 154 cm; weight: 52.2 kg; body mass index: 22.0 kg/m^2^) visited our department due to severe vomiting. Although hyperglycemia and hypertension had been identified upon screening at her workplace, she neglected these findings. She underwent medical examination for the vomiting at a local clinic; however, because her condition did not improve, she was referred to our emergency medical center. She had a history of appendicitis at 20 years of age, and she had undergone bilateral ovarian cystectomy for OMA at 28 years of age. She did not have any additional relevant medical or family history.

**Figure 1:**
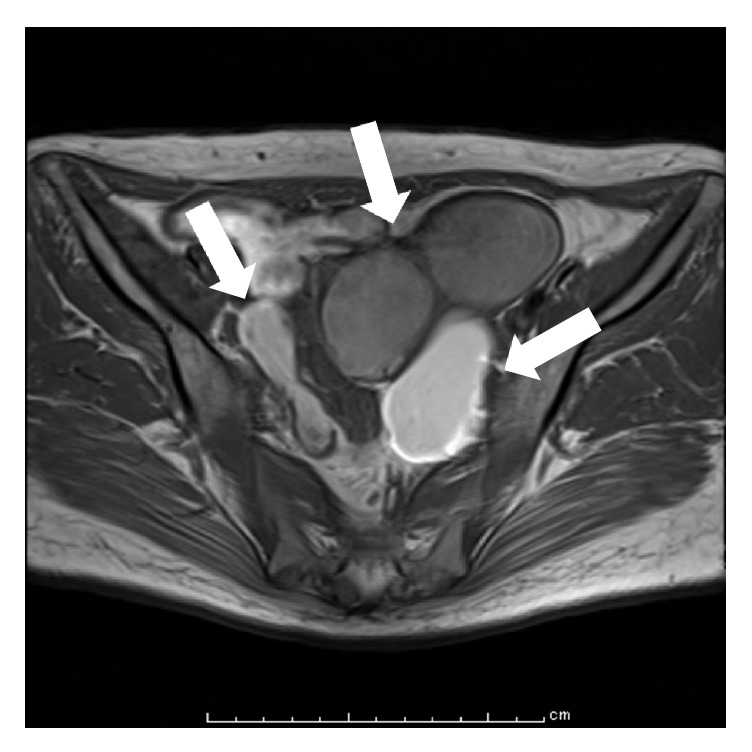
Pretreatment magnetic resonance imaging of our patient with endometriosis. Axial T1-weighted imaging at the first consultation. Two ovarian tumors (tumor size: right < left) show high-intensity signals (arrows), and the internal structure shows a blood-resistant component.

Her physical examination findings were as follows: blood pressure, 208/94 mmHg; heart rate, 96 beats/min; respiratory rate, 20 breaths/min; temperature, 36.6°C; and arterial oxygen saturation, 98%. In addition, her blood examination findings were hemoglobin level: 6.3 g/dL; hematocrit: 20.1%; white blood cell count: 17.35 × 10^3^/*μ*L; neutrophil percentage: 91.5%; platelet count: 637 × 10^3^/*μ*L; C-reactive protein level: 14.04 mg/dL; albumin level: 1.8 g/dL; blood sugar level: 450 mg/dL; HbA1c (NGSP): 13.7%; and brain natriuretic peptide level: 922.8 pg/mL. Moreover, her tumor marker findings included cancer antigen (CA) 125 level of 636.0 U/mL and CA19-9 level of 610.0 U/mL. Furthermore, her blood gas analysis findings were pH, 7.490; pCO_2_, 34.0 mmHg; and pO_2_, 64.9 mmHg. Chest radiography indicated a cardiothoracic ratio of ≤ 50% and a small pleural effusion. T1- and T2-weighted magnetic resonance images (MRI) revealed high-intensity signals in two ovarian tumors (tumor size: right < left) and masses with a maximum diameter of 59 mm in the left ovary (Figure 1). Notably, positron emission tomography-computed tomography (PTT/CT) did not show abnormal uptake. No clear malignant lesions were observed with MRI and PET/CT findings.

She was diagnosed with heart failure, type 2 diabetes mellitus, hypertension, hypoalbuminemia, and iron deficiency anemia at the initial assessment. In addition, OMA and adenomyosis of the uterus were indicated on CT. Consequently, she underwent conservative treatment without undergoing surgery since her overall status was poor.

Cyclic therapy (Gn-RH agonist, leuprolide acetate 1.88 mg, or goserelin acetate 1.8 mg via subcutaneous injection six times every 4 weeks for 24 weeks; DNG, 2 mg/day, oral administration 24 - 108 weeks) was continued alternately for approximately eight years as endometriosis treatment. Moreover, regular medical examination for tumor markers and ultrasonography was performed every three months.

**Figure 2:**
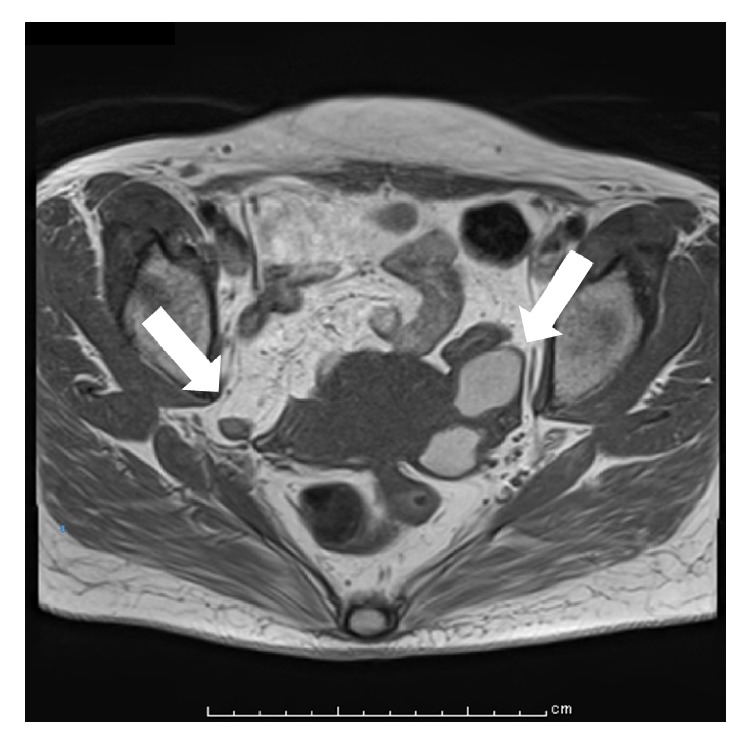
Posttreatment magnetic resonance imaging of our patient with endometriosis. Axial T1-weighted imaging after seven years of treatment. The two tumors show high-intensity signals with a diameter of 35 mm or less (arrow), and the tumor size was a partial response.

After approximately seven years of treatment, MRI findings revealed high-intensity signals in two ovarian tumors with a diameter of 35 mm or less, and the tumor size indicated a partial response (Figure 2). Moreover, the level of tumor marker had decreased.

**Figure 3:**
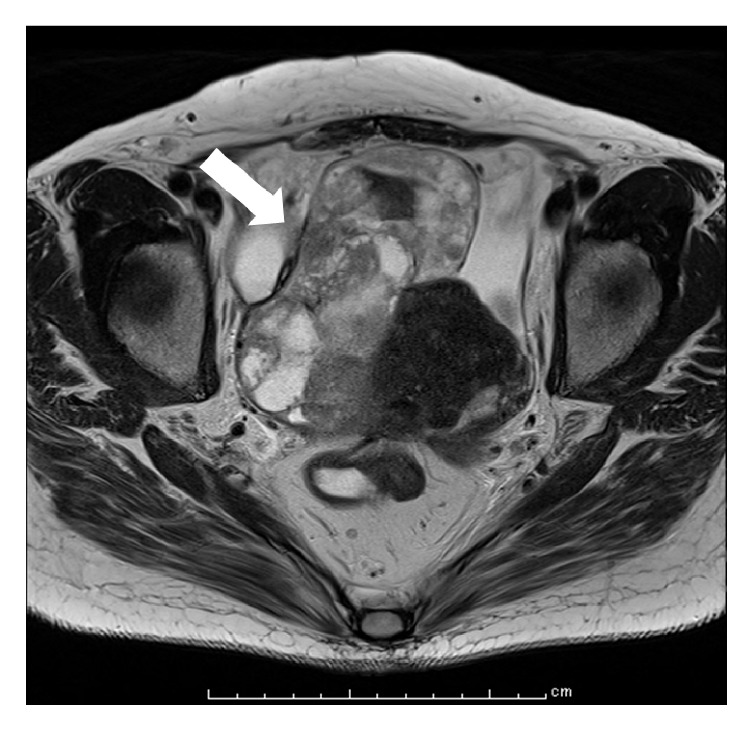
Posttreatment magnetic resonance imaging of our patient with endometriosis. MRI after eight years of treatment. The right ovary shows a mass with a maximum diameter of 114 mm (arrow). The solid part of the mass exhibits a low-intensity signal.

**Figure 4:**
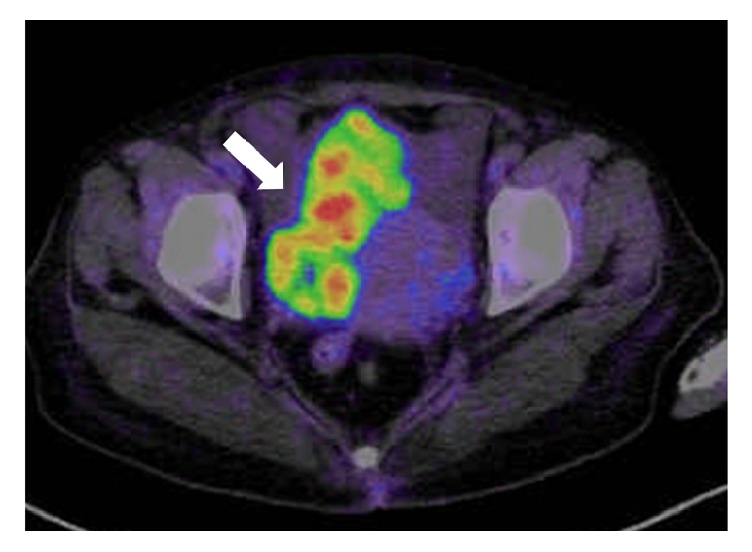
Posttreatment magnetic resonance imaging of our patient with endometriosis. PET/CT after eight years of treatment. Increased focal fludeoxyglucose accumulation (SUV max = 10.00) is observed in the solid elements of the right ovary (arrow).

**Figure 5:**
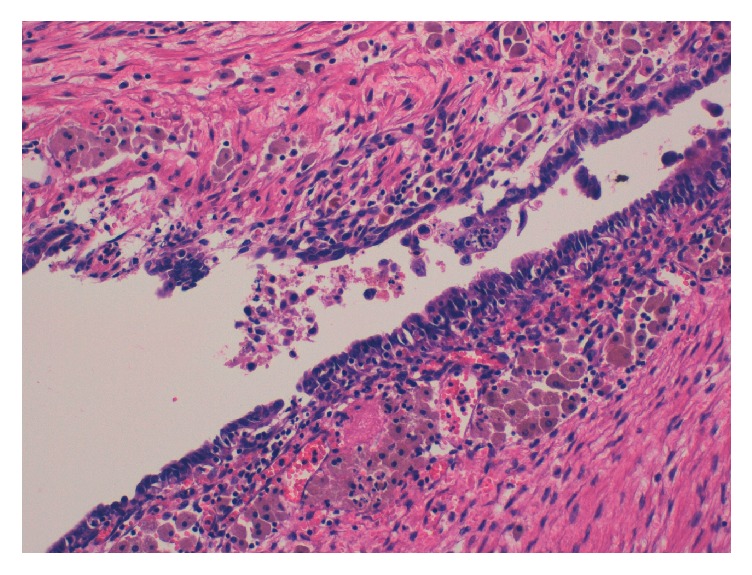
Pathological findings of the right ovary. Ovarian endometriosis of the right ovary. (H-E ×20). Ectopic endometrial tissues exist in the cyst wall, and macrophages with hemosiderin are observed.

**Figure 6:**
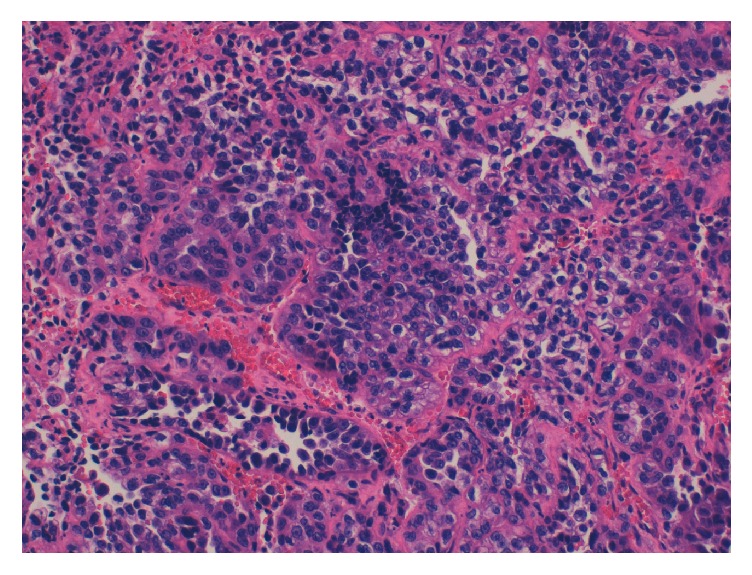
Pathological findings of the right ovary: clear cell carcinoma of the right ovary (H-E ×20) and proliferation infiltration of hobnail-like cells with pale eosinophilic cytoplasm.

However, she reported lumbago and underwent careful medical examination at our hospital after approximately eight years of treatment. Malignant transformation of the right ovarian tumor was suspected on ultrasonography (tumor enlargement and a solid mass). MRI findings revealed a mass with a maximum diameter of 114 mm in the right ovary. The solid part of the mass exhibited a low-intensity signal (Figure 3). PET/CT findings showed increased focal fludeoxyglucose accumulation (SUV max = 10.00) in the solid elements of the right ovary (Figure 4). Tumor marker findings were as follows: 125 levels, 455.2 U/mL; and CA19-9 levels, 1429.0 U/mL and surgery was performed. She underwent multiple debulking surgeries, including an abdominal total hysterectomy, bilateral salpingooophorectomy, and omentectomy. The pathological diagnosis was clear cell carcinoma of the right ovary (stage 2B [FIGO], T2b), endometriosis of the right ovary, left ovarian metastasis from right ovarian cancer, uterine infiltration from right ovarian cancer, and infiltration of clear cell carcinoma to the tissue in front of the rectum. H&E staining displayed ovarian endometriosis and clear cell carcinoma of the right ovary (Figures 5 and 6). Immunohistochemical staining results showed that the cancer cells were positive for paired box gene 8, tumor protein p53, and epithelial membrane antigen (partial positivity). Additionally, the cells were negative for the estrogen receptor, progesterone receptor, Ki-1, and Wilms' tumor suppressor gene 1.

Following surgery, she received six courses of conventional TC (paclitaxel, 175 mg/m^2^; carboplatin, AUC 5). After chemotherapy, PET/CT did not show abnormal uptake. Currently, she exhibits no evidence of disease after chemotherapy.

**Figure 7:**
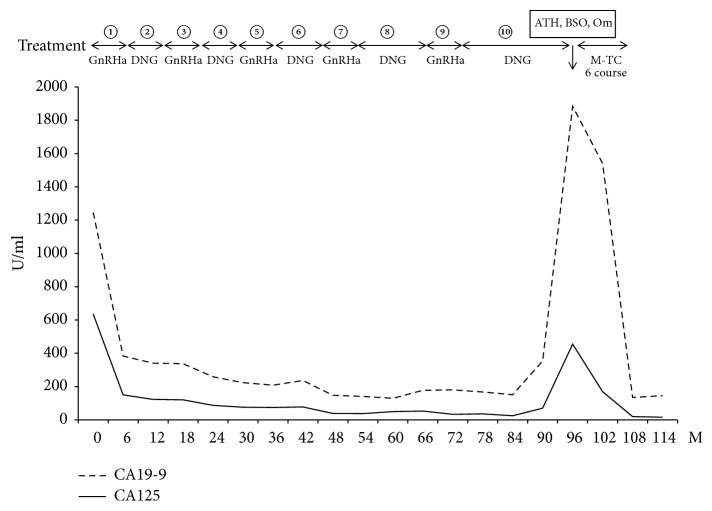
Clinical course of the patient. Increases in the level of the tumor markers, CA125 and CA19-9, are noted after six years of treatment. There was an observed decrease in the tumor markers following TC treatment.

Her clinical course is presented in Figure 7.

## 3. Discussion

OMA has a 0.5% – 1.0% probability of undergoing malignant transformation [4]. The inhibitory effect of an oral contraceptive (OC) on ovarian cancer development is remarkable in the long-term as the inhibition rates have been reported to be approximately 30%, 40%, and 50% for 5-, 10-, and 15-year OC treatments, respectively [5]. It has been demonstrated that an OC can epidemiologically prevent ovarian cancer, and the risk and reduction effects are known to correlate with the administration period [5]. An OC inhibits ovulation and appears to protect against the development of ovarian cancer by preventing major stress in the ovary [6]. DNG has both progestin effects and the ability to suppress endometriotic tissue growth, angiogenesis, and inflammation and promote apoptosis [7]. Since these mechanisms could also reduce ovarian cancer growth, we speculate that DNG may help to reduce malignant transformation of OMA [8]. The Pharmaceuticals and Medical Devices Agency (PMDA) is the government organization in Japan responsible for reviewing drugs and medical devices, overseeing post-market safety, and providing relief for adverse health effects. The PMDA reported that adverse events resulted in ovarian cancer during DNG treatment in only three cases over 10 years. In Japan, the reported cases of malignant transformation from OMA during DNG treatment are negligible [9]. Thus, malignant transformation of OMA during DNG treatment is very rare.

It has been reported that patients with diabetes mellitus have an increased risk of cancer [10, 11]. Moreover, a previous report mentioned the occurrence of ovarian cancer in patients with diabetes mellitus (n = 74 [5 with diabetes mellitus]; hazard ratio, 2.42; 95% confidence interval, 0.96–6.09) [12]. Thus, patients with diabetes mellitus in the general Japanese population may be at an increased risk of developing ovarian cancer. Since the present patient had severe diabetes, her diabetic condition might have been strongly involved in the occurrence of ovarian cancer.

In a previous pathological study on the malignant transformation of endometrioma, Heaps et al. [13] reported that a transition from endometriosis to cancer was noted in 17% of endometrioid adenocarcinoma and 24% of clear cell carcinoma cases. Moreover, Sainz et al. [14] reported that, among the cases of stage 1 ovarian cancer, endometrial carcinoma was involved in 40% of the cases, with endometrioid adenocarcinoma accounting for 41% of these cases, clear cell adenocarcinoma accounting for 31%, and mixed carcinoma (endometrioid and/or clear cell types) accounting for 18%. The transition from endometriosis to cancer is often noted in endometrioid and clear cell adenocarcinomas, and it has been suggested that endometrioma is associated with the pathogenesis of these cancers.

According to the tissue type associated with ovarian epithelial adenocarcinoma (classified by the related genes) [15], type 1 ovarian cancer includes well-differentiated serous carcinoma, well-differentiated endometrioid adenocarcinoma, clear cell adenocarcinoma, mucinous adenocarcinoma, and carcinoma from a borderline malignant tumor or chocolate cyst. Type 1 cancer causes mutations in genes, including* PTEN*,* KRAS*, and* BRAF*, and shows progressively increasing malignancy from low-grade tumors to highly differentiated adenocarcinomas. Many categories show the gradual progression to cancer over several years. Type 2 ovarian cancer includes poorly differentiated serous adenocarcinoma, poorly differentiated endometrioid adenocarcinoma, undifferentiated carcinoma, and carcinosarcoma. It is considered that most serous adenocarcinomas are derived from the fallopian tube epithelium and that many serous adenocarcinomas are accompanied by a p53 mutation and tend to show peritoneal seeding from the initial stage. Furthermore, many categories show quick progression to cancer. In the present case, the tumor could be classified as type 1 ovarian cancer, and it is presumed that OMA underwent malignant transformation over several years. In clear cell adenocarcinoma, which is considered to have a poor prognosis, dense screening is important for the early detection of ovarian malignant transformation.

The factors associated with the differential diagnosis of OMA and ovarian cancer include age, size of the ovarian cyst, tumor marker levels, and diagnostic imaging findings (i.e., ultrasonography and MRI). The risk of malignant transformation of OMA increases with age over 40 years and a maximum tumor diameter of 6 cm [16]. Serum CA125 is a typical tumor marker for endometriosis-associated ovarian carcinoma. Among patients with non-serous ovarian carcinoma (mucinous, endometrioid, and clear cell types), approximately 50% showed bordering elevation of CA125 (35 < CA125 < 65 U/mL) within a period of 3.8 years [17]. Transvaginal ultrasonography is accurate for detecting abnormalities with regard to ovarian volume and morphology; however, it is less reliable for differentiating benign ovarian tumors from malignant ovarian tumors [18]. MRI performed for clear cell adenocarcinoma typically shows a unilocular large cyst with solid protrusions, which are often round and few in number [19]. Recently, it has been shown that magnetic resonance spectroscopy might be an accurate approach for determining the total iron concentration in the cyst fluid and might represent a noninvasive method of predicting the malignant transformation of OMA [20]. This method might be clinically useful for differentiating endometriosis-associated ovarian cancer from OMA.

In conclusion, the malignant transformation of OMA is rare during DNG treatment. Since diabetes in women with endometriosis might be associated with ovarian malignant transformation, it should be carefully assessed and treated. Moreover, because a delay in the detection of ovarian cancer greatly affects prognosis, women older than 40 with OMA are encouraged undergo regular check-ups every few months.
